# The Role of HLA-G Molecule and *HLA-G* Gene Polymorphisms in Tumors, Viral Hepatitis, and Parasitic Diseases

**DOI:** 10.3389/fimmu.2015.00009

**Published:** 2015-02-02

**Authors:** Fabrício C. Dias, Erick C. Castelli, Cristhianna V. A. Collares, Philippe Moreau, Eduardo A. Donadi

**Affiliations:** ^1^Division of Clinical Immunology, Department of Medicine, Faculty of Medicine of Ribeirão Preto, University of São Paulo, Ribeirão Preto, Brazil; ^2^Department of Pathology, School of Medicine of Botucatu, Universidade Estadual Paulista, Botucatu, Brazil; ^3^Research Division in Hematology and Immunology, Institute of Emerging Diseases and Innovative Therapies, Saint-Louis Hospital, CEA, Paris, France

**Keywords:** HLA-G, tumors, viral hepatitis, parasitic disorders, polymorphism

## Abstract

Considering that the non-classical HLA-G molecule has well-recognized tolerogenic properties, HLA-G expression is expected to be deleterious when present in tumor cells and in cells chronically infected by viruses, whereas HLA-G expression is expected to be advantageous in autoimmune disorders. The expression of HLA-G on tissue or peripheral blood cells, the levels of soluble HLA-G and polymorphic sites along the gene have been studied in several disorders. In this study, we revised the role of the molecule and polymorphic sites along the *HLA-G* gene in tumors, viral hepatitis, and parasitic disorders. Overall, several lines of evidence clearly show that the induction of HLA-G expression in tumors has been associated with worse disease outcome and disease spread. In addition, the few studies conducted on hepatitis and parasitic disorders indicate that HLA-G may contribute to disease pathogenesis. Few isolated polymorphic sites, primarily located at the coding or 3′ untranslated *HLA-G* region, have been evaluated in these disorders, and a complete *HLA-G* typing together with the study of gene regulatory elements may further help on the understanding of the influence of the genetic background on disease susceptibility.

## Introduction

*HLA-G* is a non-classical class I gene of the human Major Histocompatility Complex (NCBI gene ID: 3135), presenting a restricted tissue expression pattern and encoding molecules with immune modulatory properties. This gene, firstly described by Geraghty and colleagues in 1987 (1), presents a genetic structure that resembles other classical HLA class I genes. However, contrary to that observed for classical class I genes (*HLA-A, -B*, and *-C*), the *HLA-G* gene is quite conserved among different populations and within the same population, presenting only a few non-synonymous mutations and several variation sites characterized as synonymous modifications, intronic variations, or variable sites at the regulatory regions [reviewed at Ref. (2)].

HLA-G does not seem to initiate immune responses as its classical counterparts. Instead, the HLA-G molecule is associated with the induction of inhibitory stimuli for T and B lymphocytes (3, 4), Natural Killer (NK) cells (3), and antigen-presenting cells (APC) (5). The HLA-G molecule may directly interact with multiple inhibitory receptors, including ILT2/CD85j/LILRB1 (ILT2), ILT4/CD85d/LILRB2 (ILT4), and KIR2DL4/CD158d (KIR2DL4).

The HLA-G molecule was firstly detected at the trophoblast in the maternal fetal interface, probably modulating the maternal immune system during pregnancy. Beyond trophoblast expression, HLA-G has been detected in few normal tissues, including cornea (6), thymus (7), and erythroid and endothelial precursors (8), and its upregulation has been detected in several pathological conditions as described in the present review.

Alternative splicing is also an important characteristic of the *HLA-G* gene. It may produce at least seven protein isoforms generated by alternative splicing of the primary transcript [reviewed at Ref. (2)], in which four isoforms are membrane-bound and three isoforms are soluble due to the lack of a transmembrane domain.

**Figure 1:**
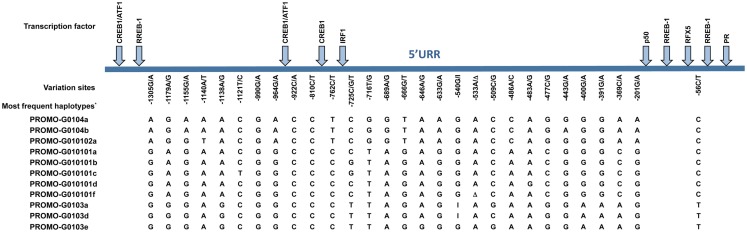
**Variation sites at the 5′ upstream regulatory region (5′URR) of the *HLA-G* gene (1.4 kb upstream of ATG), as well as the target binding sites of the described transcriptional factors**. The position of the variation sites is determined in relation to Adenine of the initiation codon ATG. *Since there is no official nomenclature for 5′URR haplotypes, they were designed as previously reported (10). Transcription factors: CREB1, CAMP responsive element binding protein 1; ATF1, cyclic AMP-dependent transcription factor ATF-1; RREB1, Ras responsive element binding protein 1; IRF1, interferon regulatory factor 1; p50, nuclear factor NF-κ-B p105 subunit; RFX5, DNA-binding protein RFX5 (RFX family); PR, progesterone receptor. I, insertion of a guanine at position −540; Δ, deletion of an adenine at position −533.

**Figure 2:**
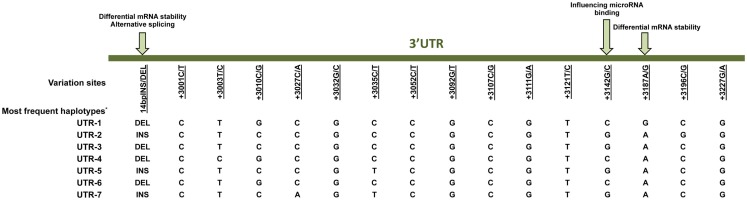
**Variation sites at the 3′ untranslated region (3′UTR) of the *HLA-G* gene that may influence HLA-G expression**. Polymorphic sites associated with diseases presented in this review are underlined. Arrows indicate polymorphic sites that have been functionally studied. *Since there is no official nomenclature for 3′UTR haplotypes, they were designed as previously reported (11). DEL, deletion; INS, insertion.

**Figure 3:**
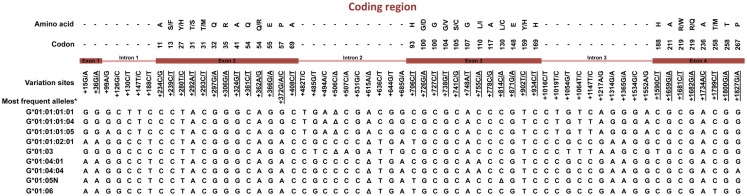
**Variation sites at the coding region of the *HLA-G* gene from exon 1–4**. Polymorphic sites associated with diseases presented in this review are underlined. *Haplotypes presenting a global frequency higher than 1% in worldwide populations. Amino acids: A, alanine; S, serine; F, phenylalanine; Y, tyrosine; T, threonine; M, methionine; Q, glutamine; R, arginine; E, glutamic acid; P, proline; H, histidine; G, glycine; D, aspartic acid; V, valine; C, cysteine; L, leucine; I, isoleucine; W, tryptophan. Δ, deletion.

Much effort has been made to evaluate *HLA-G* worldwide variability. The *HLA-G* gene seems to present functional polymorphisms mainly in the regulatory regions, probably influencing its expression. Considering data from at least 18 different populations (9–12) the *HLA-G* locus presents few frequent extended haplotypes. These haplotypes are a combination among a small number of very divergent promoter and 3′ untranslated region (3′UTR) haplotypes (Figures 1 and 2), and a coding allele usually encodes the same HLA-G molecule (Figure 3). The regulatory segments are characterized by the occurrence of several polymorphic sites presenting high heterozygosis. Although there is no consensus regarding where the *HLA-G* transcription starts (13), the polymorphisms at the 5′ upstream regulatory region (5′URR) have been considered to influence HLA-G expression, mainly because of the fact that polymorphic sites coincides with, or are close to, known transcription factor binding sites (Figure 1) [Reviewed at Ref. (13)]. Likewise, haplotypes at the *HLA-G* 3′UTR segment have been considered influencing HLA-G expression, mainly because the fact that some polymorphic sites (such as the one at position +3142) may influence the binding of specific microRNAs (14–17) or may influence mRNA stability (such as the one at position +3187) and alternative splicing (such as the 14-bp polymorphism) (Figure 2).

The *HLA-G* coding region presents mainly synonymous or intronic variation sites. Considering the most frequent *HLA-G* coding haplotypes found worldwide [reviewed at Ref. (2, 18)], only five different HLA-G full-length molecules are frequently found, in which four are complete molecules encoded by the *HLA-G*-*01:01, *01:03, *01:04, and *01:06 allele groups, and one is a truncated molecule encoded by the HLA-G*01:05N null allele. Although some different HLA-G molecules were detected worldwide, they are usually quite rare and the same *HLA-G* coding alleles are usually detected in every population studied so far. Apparently, all these frequently found molecules (exception made to the G*01:05N) present the same modulatory effects described earlier (2). Considering that only a few extended haplotypes are usually found, and considering that most of the *HLA-G* coding alleles are associated with only one promoter or 3′UTR haplotype, it is possible that most of the associations described so far regarding *HLA-G* coding polymorphism and pathological conditions are reflecting the presence of specific promoter and 3′UTR sequences and specific HLA-G production capabilities.

In the present review, we report some diseases that have been associated with the modulation of the HLA-G expression, with the presence of specific *HLA-G* gene variation sites or both, and whenever known, the mechanisms underlying such associations are discussed.

## Tumors

The arisen of transformed cells and the spread of cancer cell clones are usually controlled by the immune system cells, particularly by the action of cytototoxic T and NK cells; however, cancer cells have developed several strategies to evade host immune surveillance. Since classical histocompatibility (HLA-A, -B, and -C) molecules present tumor antigens to cytotoxic T cells, tumor cells have developed strategies to escape the cytotoxic effect of T cells by interfering with the expression of these molecules on tumor cell surface. On the other hand, the absence of HLA classical molecules on the surface of tumor cells triggers NK cell activity to eliminate neoplastic cells. If tumor cell expresses HLA-G, the cytotoxic activity of both T and NK cells are inhibited, facilitating tumor cell spread. When the decreased expression of classical HLA molecules is accompanied by an increased expression of immunomodulatory molecules such as HLA-G, the effective cytototoxic immune response against tumor cells is much impoverished [reviewed at Ref. (2)].

Although the study of HLA-G expression in tumor cells has been widely explored [reviewed at Ref. (19–21)], the evaluation of the *HLA-G* gene polymorphic sites has not been studied at the same extent, and even rarer are the studies evaluating the relationship between HLA-G tumor expression and *HLA-G* polymorphic sites. Next, we highlight some peculiarities of tumors, for which HLA-G expression (tissue or soluble levels), gene polymorphisms, or both have been evaluated.

### HLA-G expression in tumors

Increased HLA-G expression has been observed in different tumor types, including breast cancer (22–29), hepatocellular carcinoma (30–33), papillary thyroid carcinoma (34, 35), follicular thyroid carcinoma (35), follicular adenoma (35), nasopharyngeal carcinoma (36), neuroblastoma (37), bladder transitional cell carcinoma (TCC) (38), melanoma (39–42), colorectal cancer (43–45), gastric cancer (46–48), esophageal carcinoma (49–53), lung cancer (49, 54–57), renal cell carcinoma (58–62), glioblastoma (63–66), acute myeloid leukemia (67, 68), and B-cell chronic lymphocytic leukemia (69–73). Table 1 summarizes the HLA-G expression in many types of tumors described in this review.

**Table 1:** **Association between HLA-G expression and tumors**.

Tumor	HLA-G molecule				Reference
	*n*	Expression (%)	Metastasis^a^	sHLA-G (*n*)	
Breast cancer	36	36^IHC^	nd	nd	(22)
	46/39	26^(E)IHC^/41^(S)IHC^	No	nd	(74)
	58	70.7^IHC^	nd	↑(92)^ELISA^	(23)
	235	66^IHC^	Yes	↑(44)^ELISA^	(24)
	677	60^IHC^	No	nd	(27)
	nd	nd	nd	↑(45)^ELISA^	(25)
	38	58^IHC^	nd	nd	(28)
	nd	nd	nd	↑(120)^ELISA^	(75)
	52	59.6^IHC^	No	nd	(29)
	45	62^IHC^	Yes	nd	(26)

Hepatocellular carcinoma	173	57^IHC^	nd	nd	(30)
	219	50.2^IHC^	nd	↑(19)^ELISA^	(31)
	36	66.7^WB^	nd	↑(36)^ELISA^	(32)
	nd	nd	nd	↑(80)^ELISA^	(33)

Thyroid cancer	nd	nd	nd	↑(183)^ELISA^	(76)
	70	44.3^IHC^	Yes	nd	(34)
	72	77.5^IHC^	No	nd	(35)

Nasopharyngeal carcinoma	552	79.2^IHC^	Yes	nd	(36)

Neuroblastoma	12	0^IHC^	nd	↑(53)^ELISA^	(37)

Bladder transitional cell carcinoma	75	68^IHC^	nd	Ø(15)^ELISA^	(38)

Melanoma	nd	nd	nd	↑(190)^ELISA^	(39)

	79	28^IHC^	nd	nd	(40)
	35	34.2^IHC^	nd	nd	(42)

Colorectal cancer	39	87^RT-PCR^	nd	nd	(43)
	201	64.6^IHC^	Yes	nd	(44)
	nd	nd	nd	↑(144)^ELISA^	(77)
	nd	nd	nd	↑(37)^ELISA^	(49)
	251	20.3^IHC^	nd	nd	(45)

Gastric cancer	160	71^IHC^	Yes	nd	(46)
	179	49.7^IHC^	Yes	↑(179)^ELISA^	(47)
	nd	nd	nd	↑(28)^ELISA^	(49)
	52	31^IHC^	Yes	nd	(48)

Esophageal carcinoma	121	90.9^IHC^	Yes	nd	(52)
	79	65.8^IHC^	nd	↑(41)^ELISA^	(50)
	nd	nd	nd	↑(58)^ELISA^	(49)
	60	75^IHC^	No	nd	(53)
	60	70^IHC^	Yes	↑(60)^ELISA^	(51)

Lung cancer	39	26^IHC^	nd	nd	(56)
	106	75^IHC^	Yes	nd	(57)
	101	41.6^IHC^	nd	↑(91)^ELISA^	(54)
	nd	nd	nd	↑(137)^ELISA^	(55)
	nd	nd	nd	↑(43)^ELISA^	(49)

Renal cell carcinoma	18	61^IHC^	nd	nd	(59)
	38	76^qPCR^	nd	nd	(61)

Clear cell renal carcinoma	12	58^IHC^	nd	nd	(60)
	95	46.8^IHC^	nd	↑(16)^ELISA^	(62)

Glioblastoma	5	80^IHC^	nd	nd	(63)
	26	≥58^IHC^	nd	nd	(64)
	39	64^IHC^	nd	nd	(65)
	108	60.2^IHC^	nd	nd	(66)

Acute myeloid leukemia	nd	nd	nd	↑(75)^ELISA^	(78)
	77	45^FC^	nd	nd	(67)
	22	68.2^FC^	nd	nd	(68)

B-cell chronic lymphocytic leukemia	47	1–54^FC^	nd	nd	(69)
	20	1–34^FC^	nd	nd	(72)
	30	35.31^FC^	nd	nd	(73)

*^a^Association between HLA-G expression and metastasis*.

*sHLA-G, soluble HLA-G; IHC, imunohistochemistry; nd, not determined; (E), breast carcinoma effusions; (S), breast carcinoma solid lesions; ↑, increased sHLA-G levels in patients; ELISA, enzyme-linked immunosorbent assay; WB, western blotting; Ø, similar sHLA-G levels between patients and controls; RT-PCR, reverse transcriptase-PCR; qPCR, quantitative PCR; FC, flow cytometry*.

In most tumors, the increased HLA-G expression has been associated with advanced disease stages, shorter survival time, presence of metastasis, higher tumor grade, weak host immune response, greater tumor size, tumor recurrence, tumor invasion, poor histological grade, lower classical HLA antigen expression, presence of infiltrating T regulatory cells, cancer progression, increased inflammatory cell lesion infiltration, and tumor differentiation (23, 24, 26, 29–32, 34–36, 40–42, 44, 46–48, 50–54, 56, 57, 66, 69, 72, 73, 79). In other tumors, no association between increased HLA-G expression and clinicopathological features has been observed, including bladder TCC (38) and acute myeloid leukemia (67, 68).

Furthermore, increased sHLA-G levels have been reported for breast cancer (23–25, 75), hepatocellular carcinoma (31–33), papillary thyroid carcinoma (76), neuroblastoma (37), melanoma (39), colorectal cancer (49, 77), gastric cancer (47, 49), esophageal carcinoma (49–51), lung cancer (49, 54, 55), renal cell carcinoma (62), and acute myeloid leukemia (78). Higher sHLA-G levels have been associated with: (i) increased number of CD4^+^ regulatory T (Treg) cells in breast cancer (23), (ii) more aggressive tumor behavior in papillary thyroid carcinoma (76), (iii) local or disseminated relapse in neuroblastoma (37), (iv) advanced stages of disease and tumor load in melanoma (39), (v) higher IL-10 production in esophageal carcinoma (51), (vi) absence of anterior myelodysplasia along with higher leukocytosis in acute myeloid leukemia (78), and (vii) shorter survival time, high-grade tumors, higher IL-10 production, and loss of HLA classical class I molecules in patients with lung cancer (54–56).

Interestingly, sHLA-G levels were significantly decreased in breast cancer patients at 6 and 12 months after surgery (25). In addition, no association between higher sHLA-G levels and clinicopathological features has been observed in hepatocellular carcinoma (33), colorectal cancer (77), gastric cancer (47), esophageal carcinoma (50, 51), and renal cell carcinoma (62). On the other hand, plasma sHLA-G levels were closely similar when bladder TCC patients and healthy controls were compared (38).

Overall, several laboratory (increased HLA-G tumor expression, increased sHLA-G levels, increased levels of IL-10, and a cytokine that induces HLA-G expression) and clinical (advanced disease stages, worse prognosis, and presence of metastasis) findings do corroborate the malefic role of HLA-G in cancer disorders.

### Polymorphic sites at *HLA-G* gene and tumors

Several isolated segments of the *HLA-G* gene have been studied in tumors, highlighting the 3′ untranslated and coding regions. Certainly, the 14-bpINS/DEL polymorphism is the most studied. In breast cancer patients, the 14-bpDEL allele and 14-bpDEL/DEL genotype were associated with susceptibility to breast cancer in Southeastern Iranian (80) and Korean patients (81); however, no association has been reported for Brazilians (26). In addition, Korean patients exhibiting the 14-bpINS/INS genotype exhibited no HLA-G expression in breast cancer lesions (81). A meta-analysis evaluating the role of the 14-bpINS/DEL polymorphism in breast cancer reports an overall cancer risk in Asian populations (82).

The 14-bpDEL allele was associated with susceptibility to hepatocellular carcinoma in Brazilian (83) and Chinese (84) patients, but not in Korean patients (84). In addition, Chinese patients exhibiting the 14-bpDEL/DEL genotype presented increased HLA-G expression in hepatocellular carcinoma specimens (84). The 14-bpINS/DEL genotype was associated with decreased risk for childhood neuroblastoma development in Australian and New Zealand patients (85). The *HLA-G* 3′UTR haplotype known as UTR-3 (86) was associated with susceptibility to acute myeloid leukemia development in Italian patients (68).

Considering the *HLA-G* coding segment, the +755C/A (non-synonymous Leu/Ile substitution at codon 110, which defines the HLA-G*01:04 protein group) was associated with protection against more severe nasopharyngeal carcinoma tumor stages (87).

Regarding the bladder TCC, the HLA-G*01:04:04 allele, and the HLA-G*01:04 allelic group were associated with susceptibility to bladder TCC in smoking patients and the HLA-G*01:03 allele and the HLA-G*01:04 allelic group was associated with protection against bladder TCC development in non-smoking Brazilian patients. In addition, the HLA-G*01:01 allelic group and HLA-G*01:01/G*01:01 genotype were associated with susceptibility to bladder TCC development in non-smokers. Considering the bladder TCC progression, the following associations were observed: (i) the HLA-G*01:03 allele was associated with high-grade tumors among smokers; (ii) the HLA-G*01:01:01/G*01:01:02 genotype was associated with protection against high-grade tumors in the whole group of patients, whereas the same association was observed with the HLA-G*01:01 group, but only among smokers; and (iii) the HLA-G*01:04 allele group was associated with high-grade tumor development in smoker and in the whole group of patients (88).

No association has been observed for: (i) *HLA-G* coding region alleles in South Korean and Brazilian breast cancer patients (81, 89); (ii) 14-bpINS/DEL polymorphism in Italian patients presenting thyroid cancer (76); (iii) HLA-G*01:03 allele and HLA-G*01:05N null allele in Tunisian patients with nasopharyngeal carcinoma (87); (iv) HLA-G*01:05N null allele with susceptibility to esophagus carcinoma development in Chinese patients (90); (v) 14-bp INS/DEL polymorphic site in Brazilian bladder TCC patients (88); and (vi) +292A/T, +755C/A, and +1799C/T in Australian and New Zealand childhood neuroblastoma patients (85).

To date, *HLA-G* polymorphisms have not been investigated in the context of melanoma, glioblastoma, colorectal cancer, gastric cancer, lung cancer, and renal cell carcinoma.

Although some polymorphic sites (14-bpDEL allele) and coding region allele groups (HLA-G*01:04) have been previously associated with increased sHLA-G levels, few convincing associations have been reported, exception made to breast cancer for which an extensive meta-analysis has evidenced the role of this polymorphic site in Asiatic patients. Since several polymorphic sites have been described at the *HLA-G* regulatory regions, exhibiting putative roles on HLA-G expression, the typing of the complete gene and the study of the regulatory elements (transcription factors and microRNAs) produced in the tumor environment may the helpful to understand the mechanisms of tumor evasion mechanisms.

## Viral Hepatitis

Similar to tumor cells, viruses have also developed several strategies to evade the cytotoxic effect of immune effector cells, including downregulation of HLA classical class I molecules and the upregulation of non-classical molecules, or both. As a corollary, the increased HLA-G expression, induced by the virus itself or by the presence of an inflammatory milieu containing transcription and post-transcription factors that positively modulate *HLA-G* expression, may exacerbate virus morbidity and/or patient mortality. The influence of HLA-G has been studied in several chronic viral infections; some of them associated with neoplastic transformation, including human immunodeficiency virus (HIV), human papillomavirus (HPV), human cytomegalovirus (hCMV), and hepatitis viruses [reviewed at Ref. (2)].

Increased HLA-G hepatocyte expression in HCV-infected liver specimens has been associated with milder stages of fibrosis and hemosiderin deposit (91). Besides hepatocytes, HLA-G expression was observed on mast cells present in areas of liver fibrosis (92). Increased plasma sHLA-G levels were associated with chronic HCV infection and with increased IL-10 and IFN-γ levels (93). Since the treatment of mast cells with IL-10 and class I interferons induces HLA-G expression (92), infiltrating cells may play an important role on the maintenance of chronic infection and induction of chronic complications.

One study has associated increased HLA-G expression in hepatocytes with the HBV viral load (94). Different studies associated the increased serum/plasma sHLA-G levels with hepatitis B virus infection (33, 95, 96), which were associated with increased percentage of CD4^+^CD25^+^FoxP3^+^ T regulatory and HLA-G^+^CD14^+^ monocytes cells in patients exhibiting acute or chronic hepatitis (95), active hepatitis B virus infection (33) and HBeAg negative hepatitis, hepatocellular carcinoma, and increased alanine aminotransferase levels (96).

Regarding the typing of *HLA-G* 3′UTR polymorphic sites in HCV- and HBV-infected patients, the +3142C allele and 14-bpDEL/+3142C haplotype were underrepresented in Brazilian HCV-infected patients presenting sickle cells disease compared with HCV-negative group (97). On the other hand, the 14-bpINS/INS genotype was overrepresented in African-Brazilian HIV^+^ patients co-infected with HCV (HIV^+^/HCV^+^) compared with HIV^+^/HCV^−^ patients. Regarding the *HLA-G*+3142 C/G and 14-bp INS/DEL variants, no significant association has been reported for HIV^+^/HCV^+^- (98) and HBV-infected patients (99), respectively, when compared with their respective controls.

Considering that many viruses have developed evasion strategies that are similar to cancer cells and considering that many chronic viral disorders have been associated with cell transformation and malignancy, the expression of HLA-G in these disorders may predict a worse outcome and greater susceptibility to cell transformation.

## Protozoan Parasite Infections

### Human malaria infection

*Plasmodium* spp. is the etiologic agent of the human malaria and little is known about the role of HLA-G during malaria infection, and all studies have been performed to understand the mother to child transmission. One study reported a decreased HLA-G expression in extravillous trophoblast of *Plasmodium falciparum*-infected placentas compared to uninfected placentas. If by one hand, HLA-G molecule is almost exclusively expressed in extravillous trophoblast of healthy placenta specimens, on the other hand, HLA-G is detected in intervillous space macrophages of *Plasmodium*-infected placentas. In addition, NK cells are increased in infected compared to uninfected placentas (100). Furthermore, increased cord plasma levels of sHLA-G have been associated with low birth weight and increased risk of *P. falciparum* infection in infancy (101).

A family based association study performed on individuals from Niakhar, Senegal, reported that the +3187G allele was associated with higher transmission to children and lower level of parasite density during asymptomatic *P. falciparum* infection. The *HLA-G* 3′UTR haplotype known as UTR-1 was associated with a decreased level of parasite density during asymptomatic infection under a dominant model, whereas the *HLA-G* UTR-3 haplotype was associated with an increased level of parasite density during the follow-up and increased intensity of asymptomatic infection under a recessive model (102).

A second family based association study also conducted on Senegalese population has tested the association of *HLA-G* 3′UTR variants with acquired anti-malarial humoral immunity. The +3010G and +3142C alleles were overtransmitted to children with increased total IgG and IgG1 antibodies levels against glutamate-rich protein (GLURP) of *P. falciparum*, and the +3196G allele had a preferential transmission to children with a lower IgG3 response against merozoite surface protein 2 (MSP2). The *HLA-G* 3′UTR-2 haplotype was associated with a decreased IgG3 response against MSP2, suggesting a role of HLA-G on the regulation of immune humoral response during *P. falciparum* infection (103).

### Human African trypanosomiasis

Human African trypanosomiasis, also known as sleeping sickness, is caused by protozoan parasites of the *Trypanosoma brucei* species. Although no studies are available regarding HLA-G expression, genetic studies report associations of *HLA-G* gene single nucleotide variation sites with the disease. A family based association study reported that the *HLA-G* 3′UTR-14-bpINS and +3196G alleles had a preferential transmission from heterozygote parents to children and were associated with susceptibility to human African trypanosomiasis (HAT) development. In contrast, the *HLA-G* 3′UTR +3003C, +3010G, and +3187G alleles showed lower transmission from parents to children and were associated with decreased risk of developing the disease. Regarding *HLA-G* 3′UTR haplotypes, UTR-2 and UTR-5 haplotypes were associated with higher susceptibility to HAT development, whereas the *HLA-G* UTR-4 haplotype was associated with decreased risk for HAT development (104).

### American trypanosomiasis

The parasite *Trypanosoma cruzi* is the etiologic agent of American trypanosomiasis, also known as Chagas disease (105). In the chronic phase, four major clinical forms are observed: (i) cardiac that presents progressive congestive heart failure, various cardiac arrhythmias, thromboembolic events, and sudden death; (ii) digestive that is characterized by clinical signs of megaesophagus, megacolon, or both; (iii) cardiodigestive that comprises clinical and pathological signs of cardiac and digestive involvement; and (iv) indeterminate that develops without evident clinical and pathological signs (106). Recently, our group reported a decreased HLA-G expression on cardiac muscle and colonic cells in patients presenting cardiac or digestive clinical variants, respectively. On the other hand, no significant differences were observed regarding HLA-G expression in the esophagus of patients with digestive form when compared to non-chagasic patients.

Furthermore, we evaluated the polymorphic sites at the *HLA-G* 3′UTR region in Brazilian chagasic patients. The +3003T allele and +3003TT and +3187GG genotypes were overrepresented, whereas the +3003C allele and +3003CT, +3010GC, and +3042GC genotypes were underrepresented in symptomatic patients. In addition, the +3027CC and +3035CC genotypes, and the +3027C and +3035C alleles were associated with the digestive form of Chagas disease. Regarding *HLA-G* 3′UTR haplotypes, decreased UTR-4 and UTR-7 frequencies were associated with symptomatic patients and with the digestive form, respectively. On the other hand, UTR-13 was associated with the indeterminate variant and UTR-14 with the cardiac form (107).

Overall, studies on the association between HLA-G and parasitic disorders are still scarce and only the *HLA-G* 3′UTR has been evaluated.

## Conclusion

Considering the tolerogenic properties of HLA-G and considering the aphorism that the induced expression of HLA-G may be detrimental in tumors and chronic viral infection, the overall findings reported is this revision corroborates this idea. Noteworthy, is the induced expression of HLA-G on the surface of tumor cells, which has been associated with greater tumor morbidity, tumor progression, and spreading. In addition, in chronic viral infections associated with pre-neoplastic and neoplastic transformation. On the other hand, the repression of HLA-G expression is less well studied; i.e., the decreased expression of HLA-G in organs or conditions in which a constitutive expression of the molecule is expected. For instance, the decreased expression of HLA-G (placentas of *P. falciparum*-infected mothers or heart and colonic specimens of Chagas disease) has been associated with morbidity of the chronic parasitic infection.

Studies on the association of the *HLA-G* gene with diseases of diverse etiology have underestimated the myriad of polymorphic sites present at the various gene segments and have primarily focused on the evaluation of one or few polymorphic sites, particularly at the 3′UTR. Considering that many polymorphic sites along the *HLA-G* gene can be readily performed and analyzed, and considering the relevant role of isolated polymorphic sites or *HLA-G* haplotypes on HLA-G expression, HLA-G typing on diseases should add an additional tool on the understanding of the role of HLA-G on disease associations.

Theoretically, polymorphic sites observed along the coding region may modify the encoded protein and consequently the interaction with HLA-G receptors and the formation of HLA-G dimers that may more efficiently bind to HLA-G receptors. Thus, a particular allele and a particular molecule could provide susceptibility or protection against a disease development; however, such associations have not been strong enough to be considered a disease marker, as has been observed for the classical association between HLA-B27 and ankylosing spondylitis. On the other hand, polymorphic sites observed along the *HLA-G* promoter and 3′UTR gene segments may modify gene expression, accounting for disease morbidity. Unfortunately, few polymorphic sites along regulatory regions have extensively been evaluated regarding their function, and probably a combination of regulatory transcriptional and posttranscriptional elements may account for the final HLA-G production. Therefore, a complete gene evaluation together with the availability of transcription and protein profiles may provide light to the understanding of the mechanisms of HLA-G induction or repression in a specific disorder.

## Conflict of Interest Statement

The authors declare that the research was conducted in the absence of any commercial or financial relationships that could be construed as a potential conflict of interest. The Review Editor, Joel LeMaoult, declares that despite having co-authored a manuscript and being affiliated with the same institution as author Philippe Moreau, there has been no conflict of interest during the review and handling of this manuscript.
